# Early-onset dilated cardiomyopathy associated with a novel CRYAB variant complicated by non-sustained ventricular tachycardia: a case report

**DOI:** 10.1093/ehjcr/ytag299

**Published:** 2026-04-29

**Authors:** Vy Le Tran

**Affiliations:** University of Health Science, Vietnam National University, Linh Trung Ward, Thu Duc City, Ho Chi Minh City 75306, Viet Nam; Thong Nhat Hospital, Ho Chi Minh City 72121, Viet Nam

**Keywords:** Dilated cardiomyopathy, *CRYAB*, Ventricular tachycardia, Cardiac magnetic resonance, Genetics, Case report

## Abstract

**Background:**

Dilated cardiomyopathy (DCM) is a major cause of heart failure in young patients, with a genetic aetiology identified in up to 40% of cases. Variants in *CRYAB*, encoding the small heat shock protein αB-crystallin, are rare but increasingly recognized in inherited cardiomyopathies.

**Case summary:**

We report a 16-year-old male who presented with progressive heart failure symptoms and palpitations. Transthoracic echocardiography revealed a dilated left ventricle with severely reduced systolic function. Holter monitoring documented episodes of ventricular tachycardia. Cardiac magnetic resonance demonstrated global biventricular systolic dysfunction and focal mid-wall late gadolinium enhancement in the interventricular septum. Genetic testing identified a novel heterozygous *CRYAB* variant (p.Arg69Cys), confirmed by Sanger sequencing, together with a *PSEN2* variant of uncertain significance. The patient was treated with guideline-directed medical therapy for heart failure and underwent prophylactic implantable cardioverter–defibrillator implantation. Clinical status and left ventricular function improved during follow-up.

**Discussion:**

This case highlights the importance of integrating advanced cardiac imaging and genetic testing in young patients with unexplained DCM and malignant ventricular arrhythmias. Novel *CRYAB* variants may be associated with early-onset DCM and arrhythmic risk, influencing prognostic assessment and management.

Learning points

- Cardiac magnetic resonance with late gadolinium enhancement provides valuable diagnostic and prognostic information in non-ischaemic cardiomyopathy.
- Rare *CRYAB* variants may be associated with early-onset DCM and malignant ventricular arrhythmias, supporting the role of genetic testing in risk stratification.

## Introduction

Dilated cardiomyopathy (DCM) is characterized by ventricular dilatation and impaired systolic function and is a major cause of heart failure in young individuals. The condition may progress insidiously and arises from a heterogeneous range of aetiologies, including inflammatory, toxic, ischaemic, and inherited causes. Recent advances in cardiovascular genetics have identified multiple disease-associated variants implicated in inherited DCM, including *CRYAB*, which encodes the molecular chaperone αB crystallin involved in maintaining myocardial structural integrity.^1,2^

This case report highlights the diagnostic contribution of cardiac magnetic resonance imaging and genetic testing in a young patient with suspected inherited DCM. We describe a rare case of primary DCM associated with a novel *CRYAB* variant, demonstrating clinical and functional improvement following guideline-directed medical therapy.

## Case presentation

A 16-year-old male with no significant past medical history was admitted with progressive dyspnoea, orthopnoea, lower limb oedema, and recurrent palpitations. Symptoms had evolved over 4 months, with marked deterioration during the week preceding admission. There was no history of chest pain, fever, viral illness, or exposure to cardiotoxic agents. Family history was negative for cardiomyopathy, sudden cardiac death, or neuromuscular disease.

On examination, the patient was tachycardic (110–120 bpm) with elevated jugular venous pressure, bilateral ankle oedema, and a third heart sound. Blood pressure was 110/60 mmHg. Laboratory investigations revealed elevated NT-proBNP (494 pg/mL) in conjunction with normal renal function, electrolyte levels, thyroid function, and inflammatory markers.

**Figure 1:**
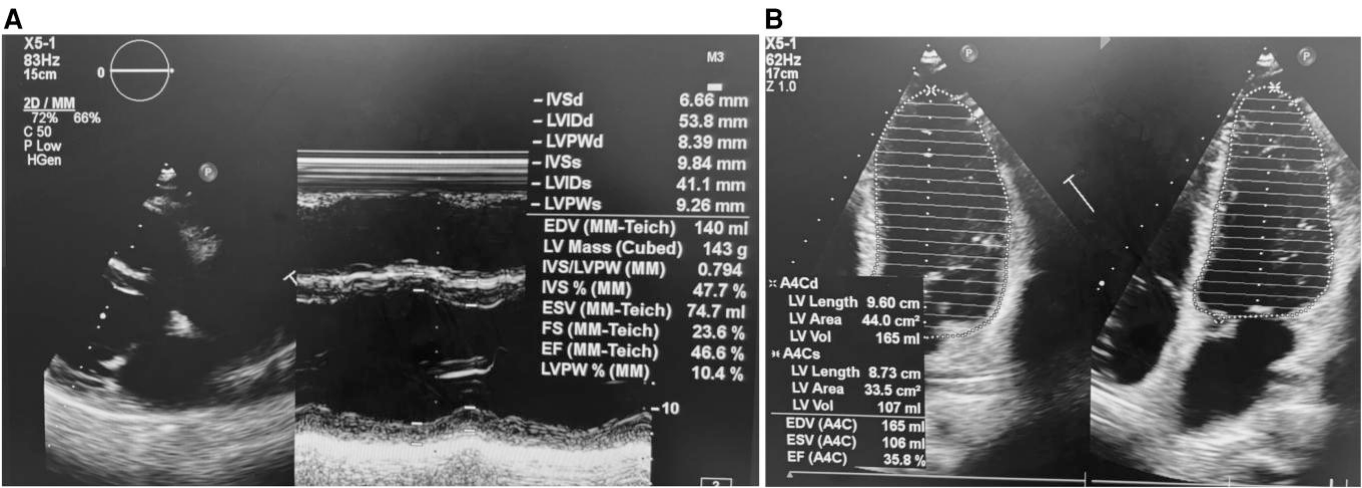
*(A)* Parasternal long-axis view with M-mode shows marked left ventricular dilatation with increased end-diastolic and end-systolic dimensions. *(B)* Apical four-chamber view demonstrates the left ventricular ejection fraction was approximately 35.8% by Simpson, consistent with severe dilated cardiomyopathy.

**Figure 2:**
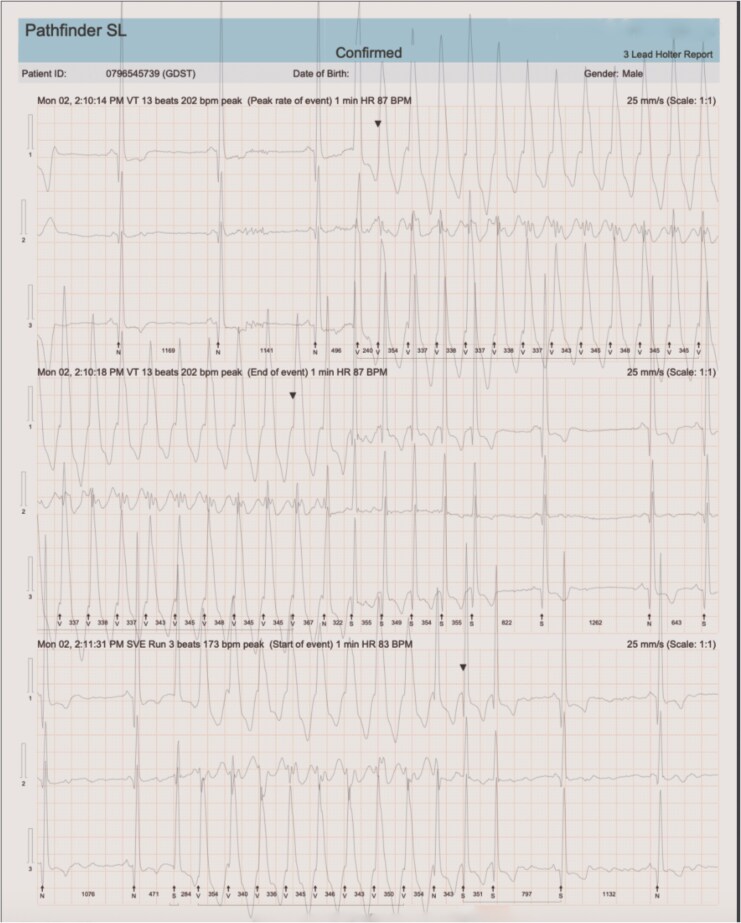
Electrocardiographic findings. The 12-lead electrocardiogram demonstrated sinus tachycardia at rest. ECG Holter monitoring demonstrated sinus rhythm, paroxysms of narrow complex tachycardia (170 bpm), and high-burden ventricular arrhythmia comprising 2853 polymorphic premature ventricular complexes (2%) and 76 non-sustained ventricular tachycardia runs.

Transthoracic echocardiography demonstrated global left ventricular dilation with reduced left ventricular ejection fraction (LVEF) of 35.8%, mild functional mitral regurgitation, moderate tricuspid regurgitation, and pulmonary hypertension (PASP 45 mmHg). Left ventricular ejection fraction was assessed using the biplane Simpson’s method in accordance with current echocardiographic recommendations (*Figure 1*). The resting ECG showed sinus tachycardia. A 24-h Holter monitor recorded non-sustained ventricular tachycardia (*Figure 2*).

**Figure 3:**
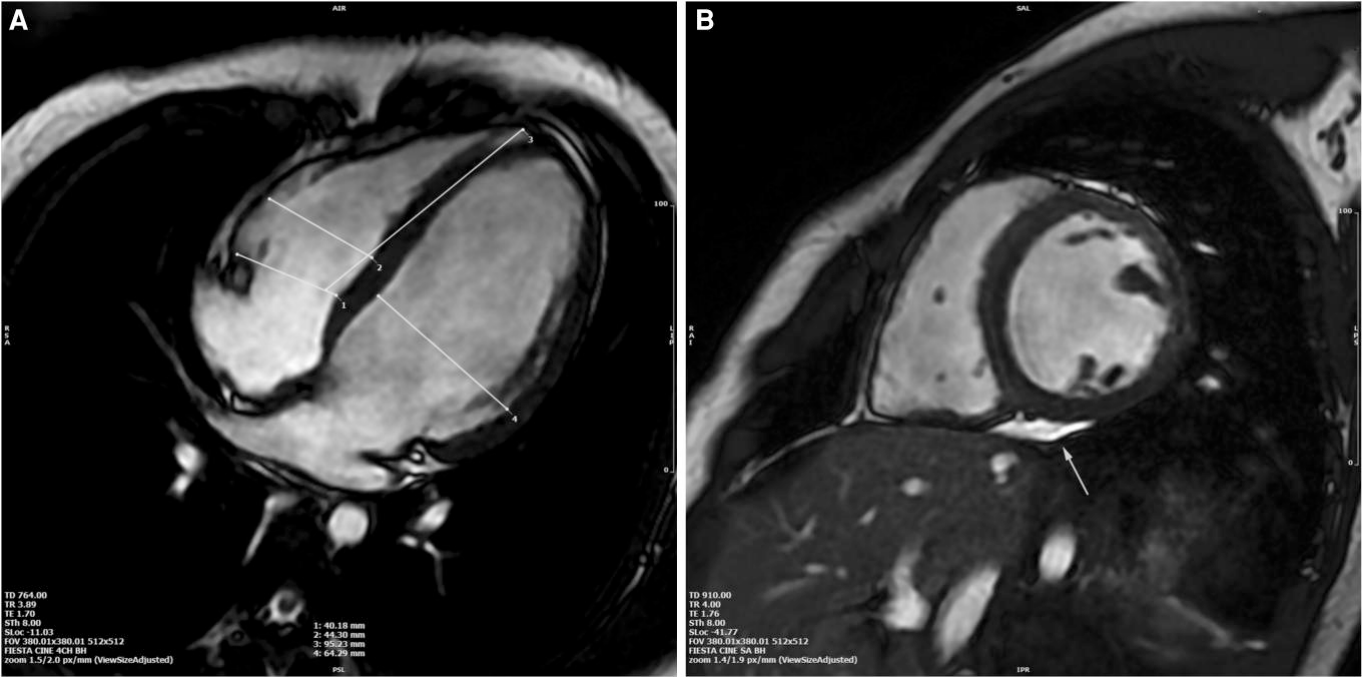
Cardiac magnetic resonance imaging. Cardiac magnetic resonance imaging demonstrated increased biventricular volumes and reduced systolic function. (*A*) showed a large four-chamber view. (*B*) The arrow highlights a focal hyperintensity adjacent to the epicardial border, which does not conform to a typical pattern of myocardial enhancement. This appearance is most consistent with an imaging artifact and should not be misinterpreted as myocardial fibrosis.

Coronary computed tomography angiography excluded obstructive coronary artery disease. Cardiac magnetic resonance revealed increased biventricular volumes, reduced LVEF (34%) and RVEF (41%), and focal mid-wall late gadolinium enhancement in the interventricular septum, accounting for approximately 1% of left ventricular mass. No features of myocarditis or infiltrative disease were identified (*Figure 3*).

Genetic testing using whole-exome sequencing (WES) identified a heterozygous *CRYAB* variant (c.205C > T, p.Arg69Cys), not previously reported in population databases, and a *PSEN2* variant (p.Ala230Thr) of uncertain significance. The *CRYAB* variant was confirmed by Sanger sequencing and classified as likely pathogenic according to ACMG criteria (*Table 1*).

**Table 1:** Timeline of multimodality imaging, aetiological work-up, and therapeutic management

Time point	Key findings	Interpretation/clinical action
**4 months before admission**	Progressive exertional dyspnoea (NYHA II→III), orthopnoea, 4-kg weight gain, bilateral lower-limb oedema	Clinical suspicion of heart failure
**At admission**	HR 110–120 b.p.m.; elevated JVP; S3; peripheral oedema. ECG: sinus tachycardia. CXR: cardiomegaly. NT-proBNP 494 pg/mL. CRP 5 mg/L. Thyroid function within reference range	Acute decompensated heart failure
**Transthoracic echocardiography**	LVEF 35.8%; dilated LV; mild RV dilatation; moderate MR; estimated PAPs 45 mmHg	Dilated cardiomyopathy with reduced ejection fraction; further aetiological evaluation indicated
**Coronary CT angiography**	No significant coronary artery stenosis	Ischaemic aetiology excluded
**Cardiac magnetic resonance**	LVEDV 154 mL/m^2^; RVEDV 108 mL/m^2^; LVEF 34%; RVEF 41%; focal mid-wall septal late gadolinium enhancement (2 g; 1% LV mass); no myocardial oedema on T2-weighted imaging	Non-ischaemic fibrosis pattern consistent with dilated cardiomyopathy; active inflammatory myocardial disease unlikely
**Genetic testing**	Variants identified in CRYAB (p.Arg69Cys) and PSEN2 (p.Ala230Thr)	Findings support a genetic non-ischaemic dilated cardiomyopathy in the appropriate clinical context
**In-hospital management**	One week of guideline-directed medical therapy	Marked clinical improvement with resolution of peripheral oedema and significant relief of dyspnoea; discharged in stable condition
**Three-month follow-up**	Clinically stable; improved exercise tolerance (NYHA I); no orthopnoea or paroxysmal nocturnal dyspnoea; LVEF unchanged from baseline	Symptomatic improvement without measurable recovery of systolic function
**Risk stratification**	HFrEF with non-sustained ventricular tachycardia and myocardial fibrosis	Prophylactic implantable cardioverter–defibrillator implantation in addition to guideline-directed medical therapy

The diagnostic evaluation required exclusion of alternative causes of left ventricular dilatation in a young patient. Acute or subacute myocarditis was considered, in line with ESC recommendations on cardiomyopathies and inflammatory myocardial disease. However, cardiac magnetic resonance showed focal mid-wall late gadolinium enhancement confined to the interventricular septum without myocardial oedema on T2-weighted imaging, arguing against active inflammation and favouring a non-ischaemic fibrosis pattern typical of DCM.^3,4^ Tachycardia-induced cardiomyopathy was deemed unlikely, as no sustained tachyarrhythmia preceded ventricular dysfunction, and non-sustained ventricular tachycardia was interpreted as secondary to structural disease. Metabolic and toxic causes were excluded based on history, laboratory evaluation, and absence of systemic involvement. Although idiopathic DCM remained a theoretical possibility, the identification of a likely pathogenic CRYAB variant, together with a fibrosis substrate on CMR, supported a primary genetic cardiomyopathy in accordance with contemporary ESC guidance.^3^

The patient was treated with guideline-directed medical therapy for heart failure with reduced ejection fraction, including sacubitril/valsartan, beta-blocker, mineralocorticoid receptor antagonist, and SGLT2 inhibitor. Given the presence of ventricular tachycardia and myocardial fibrosis, a prophylactic implantable cardioverter–defibrillator was implanted.

Following 1 week of inpatient guideline-directed medical therapy, the patient showed marked clinical improvement, with complete resolution of peripheral oedema and significant relief of dyspnoea, allowing hospital discharge. At the 3-month follow-up, the patient remained clinically stable with improved exercise tolerance, corresponding to New York Heart Association (NYHA) functional Class I. The patient reported no orthopnoea or paroxysmal nocturnal dyspnoea. Left ventricular ejection fraction at follow-up did not change significantly from baseline, indicating symptomatic improvement without measurable recovery of systolic function.

The diagnostic pathway, including multimodality imaging and genetic evaluation, is summarized in Timeline.

### Genetic analysis and ACMG classification

Genetic testing was performed using WES with a focus on genes associated with inherited cardiomyopathies and arrhythmia syndromes (*Table 2*). Sequencing achieved adequate coverage (>99% of target regions at >20× depth). Bioinformatic analysis identified a heterozygous missense variant in the *CRYAB* gene (NM_001885.3:c.205C>T; p.Arg69Cys). This variant was confirmed by Sanger sequencing. The variant was absent from large population databases, including gnomAD, and has not been previously reported in ClinVar or the published literature, supporting its novelty.^5,6^

**Table 2:** Identified genetic variants and clinical interpretation

Gene	Inheritance	Zygosity	Genomic location	Nucleotide/protein change	Variant classification	Reported associated phenotypes	Clinical significance
***CRYAB***	Autosomal dominant (AD)/autosomal recessive (AR)	Heterozygous	chr11:111910446	NM_001289808.2:c.205C>T (NP_001276737.1:p.Arg69Cys)	Missense variant	Dilated cardiomyopathy, Type III (AD); cataract 16, multiple types (AD, AR); myofibrillar myopathy 2 (AD); fatal infantile hypertonic myofibrillar myopathy, αB-crystallin-related (AR)	Functional impact not fully elucidated
***PSEN2***	Autosomal dominant (AD)	Heterozygous	chr1:226888950	NM_000447.3:c.688G>A (NP_000438.2:p.Ala230Thr)	Missense variant	Alzheimer disease-4 (AD); dilated cardiomyopathy, type IV (AD)	Not previously reported in ClinVar

Sanger sequencing confirms a heterozygous missense variant in *CRYAB* (NM_001885.3:c.205C>T; p.Arg69Cys). This novel variant was classified as likely pathogenic according to the American College of Medical Genetics and Genomics (ACMG) criteria.

The PSEN2 variant was classified as a variant of uncertain significance (VUS) according to ACMG criteria. It was considered incidental due to the absence of segregation with the cardiac phenotype, the lack of an established association with dilated cardiomyopathy, and the incidental finding in population databases. No additional cardiovascular or neurocognitive phenotypes suggestive of PSEN2-related disease were present.

AD, autosomal dominant; AR, autosomal recessive; HGVS, Human Genome Variation Society.

*In silico* predictive tools suggested a deleterious effect on protein function (PolyPhen-2: probably damaging; SIFT: deleterious; CADD score >20). The affected residue is highly conserved across species, and *CRYAB* is known to encode αB-crystallin, a small heat shock protein with an essential role in maintaining cytoskeletal integrity and proteostasis in cardiomyocytes under stress conditions. Missense variants in *CRYAB* have been previously associated with cardiomyopathy and arrhythmic phenotypes, supporting biological plausibility.^7^

According to the American College of Medical Genetics and Genomics (ACMG) guidelines, the CRYAB p.Arg69Cys variant was classified as likely pathogenic based on the following criteria: PM2 (absence from population databases), PP3 (multiple *in silico* tools predicting a deleterious effect), PP4 (phenotype particular for a disease with a single genetic aetiology), and PS4_supporting (enrichment in affected individuals compared with controls).^8^ Details of the variant interpretation are summarized in *Table 3*.

**Table 3:** ACMG classification of the CRYAB variant identified in this patient

ACMG criterion	Evidence level	Justification
**PM2**	Moderate	The variant (*CRYAB* NM_001885.3:c.205C>T; p.Arg69Cys) is absent from population databases, including gnomAD, indicating it is not a common benign variant.
**PP3**	Supporting	Multiple *in silico* prediction tools suggest a deleterious effect on protein function (PolyPhen-2: probably damaging; SIFT: deleterious; CADD score >20).
**PP4**	Supporting	The patient’s phenotype (early-onset dilated cardiomyopathy with malignant ventricular arrhythmia) is highly specific for a genetic cardiomyopathy, consistent with previously reported *CRYAB*-related disease mechanisms.
**PS4_supporting**	Supporting	The variant is extremely rare and identified in a patient with a phenotype consistent with the gene–disease association, although large case-control data are not available.

A heterozygous missense variant in *PSEN2* was also identified and classified as a variant of uncertain significance. Although *PSEN2* has been sporadically reported in association with DCM, this variant lacks supporting functional data, segregation evidence, or a consistent genotype–phenotype correlation. In the presence of a more compelling likely pathogenic *CRYAB* variant that better explains the clinical presentation, the *PSEN2* variant was therefore considered incidental.^8,9^

Family screening had not been completed at the time of reporting and represents an important limitation. The lack of segregation analysis restricts confirmation of variant pathogenicity and weakens genotype–phenotype interpretation, as intrafamilial cosegregation could have provided critical supportive evidence.

## Discussion

This case underscores the clinical value of combining advanced imaging with genetic testing in young patients with unexplained DCM. Such an approach facilitates accurate diagnosis, informs prognostic assessment, guides family screening, and influences management decisions, including consideration of prophylactic ICD implantation. Prophylactic ICD implantation was justified by the markedly reduced LVEF, documented non-sustained ventricular tachycardia, mid-wall late gadolinium enhancement on CMR, adolescent onset, and a potentially arrhythmogenic genetic substrate. Mid-wall fibrosis and NSVT are recognized markers of increased arrhythmic risk in non-ischaemic DCM, beyond LVEF alone. Current ESC guidelines support primary prevention ICD in patients with LVEF ≤35% despite optimal medical therapy, particularly when additional risk modifiers such as myocardial fibrosis or ventricular arrhythmias are present.^1,3,10^

Although CRYAB variants have previously been associated with DCM and myofibrillar myopathy, this case broadens the recognized clinical spectrum by demonstrating adolescent onset, isolated cardiac involvement, and an arrhythmogenic presentation without neuromuscular manifestations. CRYAB encodes αB-crystallin, a small heat shock protein expressed in cardiomyocytes; impairment of its chaperone function can disrupt cellular protein homeostasis under stress, leading to myocyte injury, adverse remodelling, and replacement fibrosis that may provide a structural substrate for ventricular arrhythmias.^7,11^

The identification of a novel variant in this patient further extends the clinical profile of αB-crystallin-related disease, highlighting its association with early-onset DCM and non-sustained ventricular tachycardia accompanied by high-risk features. The presence of mid-wall late gadolinium enhancement on cardiac magnetic resonance imaging supported a non-ischaemic, likely genetic aetiology and offers a mechanistic link between the underlying variant and the observed arrhythmic phenotype.^3,12^ Previously reported pathogenic or likely pathogenic alterations have predominantly been described in the context of myofibrillar myopathy with or without cardiac involvement, as well as restrictive or hypertrophic phenotypes.^2,7,12^ Reports of isolated DCM remain limited and have largely involved adult-onset or syndromic presentations with skeletal muscle disease. In addition, non-sustained ventricular tachycardia has been inconsistently documented, and data regarding arrhythmic risk in paediatric or adolescent patients are sparse. Compared with these reports, our patient presented with early-onset DCM complicated by ventricular tachycardia in the absence of neuromuscular manifestations, supporting the concept that selected variants may manifest primarily with cardiac and arrhythmogenic features.

## Patient perspective

The patient and his family expressed that genetic testing and CMR findings helped them better understand the nature of his condition and the rationale for prophylactic ICD therapy, reinforcing their trust in the treatment plan.

## Conclusion

Early-onset DCM with ventricular arrhythmias should prompt a comprehensive evaluation, including cardiac magnetic resonance imaging and genetic testing. Novel *CRYAB* variants may confer significant arrhythmic risk, and their recognition is essential for personalized management and prevention of sudden cardiac death.

## Data Availability

The data underlying this article are available within the article and its supplementary material. Genetic data are not publicly available due to ethical and privacy considerations but may be shared upon reasonable request to the corresponding author.

## Lead author biography

Dr. Vy Le Tran is a cardiologist with a clinical and research interest in inherited cardiomyopathies and cardiovascular genetics. Her work focuses on the diagnostic evaluation and risk stratification of cardiomyopathy in young patients, with particular attention to genotype-phenotype correlations and arrhythmic complications. She is actively involved in clinical practice, academic research, and medical education in the field of cardiovascular medicine.

## Notes

**Consent:** Written informed consent was obtained from the patient’s parents for the publication of this case and the accompanying images.
